# The Alkaline Phosphatase (*ALPL*) Locus Is Associated with B6 Vitamer Levels in CSF and Plasma

**DOI:** 10.3390/genes10010008

**Published:** 2018-12-22

**Authors:** Loes M. Olde Loohuis, Monique Albersen, Simone de Jong, Timothy Wu, Jurjen J. Luykx, Judith J. M. Jans, Nanda M. Verhoeven-Duif, Roel A. Ophoff

**Affiliations:** 1 Center for Neurobehavioral Genetics, Semel Institute for Neuroscience and Human Behavior, University of California Los Angeles (UCLA), Los Angeles, CA 90095, USA; loldeloohuis@mednet.ucla.edu (L.M.O.L.); sdejongwork@gmail.com (S.d.J.); Timothy.Wu@bcm.edu (T.W.); 2 Section Metabolic Diagnostics, Department of Genetics, University Medical Center (UMC), 3584 EA Utrecht, The Netherlands; m.albersen@vumc.nl (M.A.); J.J.M.Jans@umcutrecht.nl (J.J.M.J.); N.Verhoeven@umcutrecht.nl (N.M.V.-D.); 3 Department of Psychiatry, Rudolf Magnus Institute of Neuroscience, University Medical Center (UMC), 3584 CG Utrecht, The Netherlands; jjluykx@gmail.com; 4 Department of Translational Neuroscience, Human Neurogenetics Unit, Brain Center Rudolf Magnus, University Medical Center Utrecht (UMC), 3584 CG Utrecht, The Netherlands

**Keywords:** genome wide association study, vitamin B6, cerebrospinal fluid, plasma

## Abstract

The active form of vitamin B6, pyridoxal phosphate (PLP), is essential for human metabolism. The brain is dependent on vitamin B6 for its neurotransmitter balance. To obtain insight into the genetic determinants of vitamin B6 homeostasis, we conducted a genome-wide association study (GWAS) of the B6 vitamers pyridoxal (PL), PLP and the degradation product of vitamin B6, pyridoxic acid (PA). We collected a unique sample set of cerebrospinal fluid (CSF) and plasma from the same healthy human subjects of Dutch ancestry (*n* = 493) and included concentrations and ratios in and between these body fluids in our analysis. Based on a multivariate joint analysis of all B6 vitamers and their ratios, we identified a genome-wide significant association at a locus on chromosome 1 containing the *ALPL* (alkaline phosphatase) gene (minimal *p* = 7.89 × 10^−10^, rs1106357, minor allele frequency (MAF) = 0.46), previously associated with vitamin B6 levels in blood. Subjects homozygous for the minor allele showed a 1.4-times-higher ratio between PLP and PL in plasma, and even a 1.6-times-higher ratio between PLP and PL in CSF than subjects homozygous for the major allele. In addition, we observed a suggestive association with the CSF:plasma ratio of PLP on chromosome 15 (minimal *p* = 7.93 × 10^−7^, and MAF = 0.06 for rs28789220). Even though this finding is not reaching genome-wide significance, it highlights the potential of our experimental setup for studying transport and metabolism across the blood–CSF barrier. This GWAS of B6 vitamers identifies alkaline phosphatase as a key regulator in human vitamin B6 metabolism in CSF as well as plasma. Furthermore, our results demonstrate the potential of genetic studies of metabolites in plasma and CSF to elucidate biological aspects underlying metabolite generation, transport and degradation.

## 1. Introduction 

The active form of vitamin B6, pyridoxal phosphate (PLP), functions as a co-factor in >200 enzymatic reactions in human metabolism [1,2]. Inverse relationships have been found between vitamin B6 and conditions such as diabetes, oxidative stress, cardiovascular disease, inflammation and cancer [3,4,5,6,7]. Its role in amino acid and neurotransmitter metabolism renders vitamin B6 specifically essential for brain development and functioning. Lower concentrations of PLP in plasma have not only been associated with symptoms of depression (according to the Major Depression Inventory [8]), but also with poor cognition [9] and Alzheimer’s disease [10,11]. 

The dependence of the neurotransmitter balance in the brain on vitamin B6 is illustrated by inborn errors of metabolism resulting in hampered vitamin B6 metabolism. Such patients may present with epilepsy and intellectual disability [12]. Examples of these genetic disorders are antiquitin deficiency (OMIM #266100) [13], pyridox(am)ine-5′-phosphate oxidase (PNPO) deficiency (OMIM #610090) [14], hyperprolinaemia type II (pyrroline-5-carboxylate dehydrogenase deficiency; OMIM #239510) [15] and hypophosphatasia (alkaline phosphatase (ALPL) deficiency; OMIM #241500) [16,17]. Although in antiquitin and PNPO deficiencies, treatment with vitamin B6 is successful in antagonizing the epilepsy, patients with these inborn metabolic disorders will still suffer from intellectual disability [12,18]. In addition to these known causes of functional vitamin B6 deficiency, there are patients in whom the cause of their vitamin B6-responsive epilepsy remains unelucidated. 

**Figure 1:**
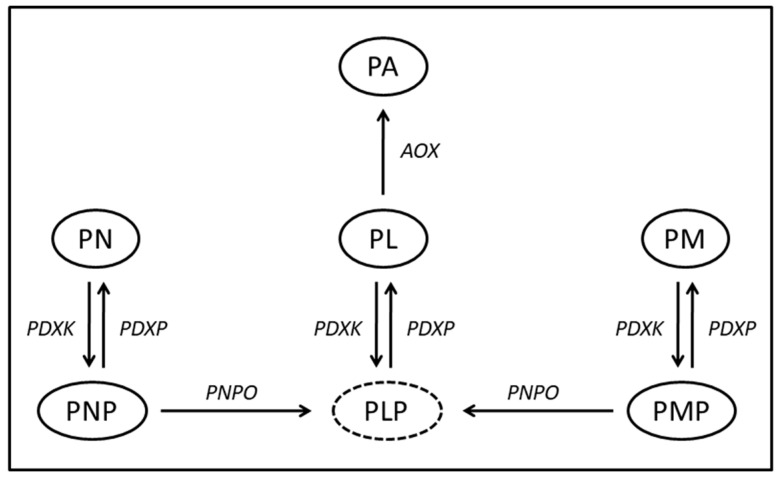
Schematic display of vitamin B6 metabolism. The different B6 vitamers pyridoxine (PN), pyridoxamine (PM), pyridoxal (PL) and their respective phosphate-esters (PNP, PMP and PLP) are interconvertible through the action of several enzymes. Pyridoxic acid (PA) is the main degradation product of vitamin B6. *PDXK* = pyridoxal kinase. *PDXP* = pyridoxal phosphatase. *PNPO* = pyridox(am)ine oxidase. *AOX* = aldehyde oxidase.

Humans depend on dietary sources of vitamin B6 since they are unable to synthesize it. The different B6 vitamers—pyridoxine (PN), pyridoxamine (PM), pyridoxal (PL) and their respective phosphate-esters (Figure 1)—are interconvertible through the action of several enzymes. Transport across the cell membrane is preceded by hydrolysis of the phosphorylated B6 vitamers by membrane-bound alkaline phosphatase (ALPL) [19]. The ALPL locus has been associated with PLP and vitamin B6 in plasma [20,21,22,23]. Intracellularly, phosphorylation by pyridoxal kinase (PDXK) [24] yields PNP, PMP and PLP (Figure 1). Pyridox(am)ine phosphate oxidase converts PNP and PMP into the active co-factor, PLP [25]. Release of vitamin B6 from the cell is dependent on a vitamin B6-specific phosphatase (pyridoxal phosphatase (PDXP)) [26]. Oxidation of the resulting PL by aldehyde oxidase (AOX) [27] constitutes the degradation pathway of vitamin B6, of which the major product, pyridoxic acid (PA), is excreted into urine (Figure 1) [28]. Although the enzymes involved in vitamin B6 metabolism have been elucidated at genetic and protein levels, knowledge about human vitamin B6 transport is very limited. At the biochemical level, there is evidence for carrier-mediated transport [29,30,31], but not a single human vitamin B6 transporter has been identified to date. 

From our previous studies [32,33], we know that the B6 vitamer composition of human cerebrospinal fluid (CSF) differs from that of human plasma, and that B6 vitamer concentrations are tightly controlled in and between both body fluids. Vitamin B6 intake, metabolism, transport and/or genetic regulation are possible mechanisms contributing to these observations. To study the genetic regulation of vitamin B6 homeostasis, we conducted a genome-wide association study (GWAS) of B6 vitamers and their ratios. We collected a unique sample set of CSF and plasma from the same 493 healthy human subjects. We aimed to explore the genetic architecture of B6 metabolism in CSF and the genetic control of the crosstalk between the central nervous system and peripheral blood. 

## 2. Subjects and Methods

### 2.1. Subjects and Sample Collection 

Subject characteristics and collection of samples have been described in detail by Luykx et al. [34,35]. In summary, plasma and/or CSF were collected from 533 healthy and fasting subjects undergoing spinal anesthesia for minor elective surgery in different hospitals in and near Utrecht, The Netherlands. Subjects were 18–63 years of age and of North-Western European descent. Most of the subjects underwent knee arthroscopy and do reflect the general population with respect to comorbidities [36]. The study was approved by the ethics committee of the University Medical Center (UMC) Utrecht and by all local ethics committees (The Medisch Ethische Toetsingscommissie approval number 23042.041.08 date 07/30/08). The participants provided written informed consent. After withdrawal, plasma and CSF were stored at −80 °C until further analysis.

### 2.2. Determination of B6 Vitamer Concentrations 

Concentrations of the B6 vitamers PL, PLP, PM, PMP and PN, as well as the concentration of PA, were determined in plasma and CSF (nmol/L) by ultra-performance liquid chromatography–tandem mass spectrometry (UPLC–MS/MS) [33,37]. After exclusion of subjects with outlier B6 vitamer concentrations (*n* = 10, e.g., B6 vitamers >1.5 times lower or higher than the lower or upper reference limit, see [33] for details), B6 vitamer concentrations in plasma and/or CSF of 523 subjects remained for genome-wide association analyses (see Table 1 for more details). Ratios between B6 vitamers in CSF and plasma, and ratios for B6 vitamers between CSF and plasma, were calculated.

### 2.3. Phenotyping

As described previously [32,33,37,38], concentrations of PMP and PN in plasma and CSF, as well as the concentration of PM in plasma, generally are below limits of quantification and thus undetectable. Since this also applied to our current dataset, these B6 vitamers were not included in the association analyses. Because the very low concentrations of PM and PA in CSF could not be reliably quantified (relatively high variation) [37], PM and PA in CSF were also not taken along in the association analyses. 

Since none of the B6 vitamers, nor their unstandardized residuals, showed a normal distribution, B6 vitamer concentrations and ratios were normalized using a rank-based inverse normal transformation (INT) [20] before association analyses were performed. 

We also included ratios between B6 vitamers and ratios between fluid types in our analyses. Ratios between the B6 vitamers constitute an approximation of the conversion rate of the enzymatic reactions of substrate–product pairs [39]. Another potential advantage of taking metabolite ratios is the signal-to-noise ratio reduction by correcting for known and unknown confounders such as hidden batch effects [40]. Additionally, the ratios between CSF and plasma may inform us on functional mechanisms of vitamin B6 transport across the blood–brain or blood–CSF barriers. 

### 2.4. Genotyping, Imputation and Quality Control Procedures 

From 506 out of 523 subjects in whom we determined plasma and/or CSF B6 vitamer concentrations, whole-genome single nucleotide polymorphism (SNP) data were available (Illumina HumanOmniExpressExome Beadchip (987,734 SNPs), UCLA Neuroscience Genomic Core facility [35]). The genotyping platform and methods have been described in detail by Luykx et al. [35].

Prior to imputation, we applied the following quality control using PLINK (v1.08p) [41]. We excluded samples with ambiguous sex or with imputed sex inconsistent with our database (*n* = 1), as well as samples with missing genotyping >2% (*n* = 9). Based on a set of 87,956 independent high-quality SNPs (with a minor allele frequency (MAF) >10%, missing genotype rate <1%, Hardy Weinberg equilibrium (HWE) *p*-value < 1.0 × 10^−5^, and a maximum linkage disequilibrium (LD) R^2^ of 0.2), we tested for too high (>mean +3SD) or too low (<mean −3SD) heterozygosity (*n* = 0). We also excluded samples related up to the level of distant cousins (pi-hat <0.2, *n* = 1), and outliers based on multidimensional scaling (MDS) clustering of our data with HapMap3 [42] (*n* = 2; Supplementary Figure S1). We removed SNPs with missingness >2%, HWE *p* < 1.0 × 10^−6^; and excluded non-autosomal SNPs. The remaining *n* = 493 samples and 903,536 variants were used for imputation. 

Imputation was performed using the Michigan imputation server [43], the 1000 G Phase3v5 reference panel, HapiUR phasing, and the European reference population. Post-imputation, we selected high-quality variants with an imputation score RSQ > 0.8 and converted the dose files to PLINK format. From the set of 9,135,989 high-quality imputed variants, we only kept variants with HWE *p* < 0.001, MAF > 0.05 in our GWAS, resulting in a total number of *n* = 493 samples and 6,260,487 SNPs to be included in our analyses. 

### 2.5. Multivariate Analysis

To detect SNPs relevant to B6 vitamer concentrations and ratios in and between CSF and plasma, we performed a genome-wide multivariate association study. 

Multivariate association studies have several advantages over performing several univariate analyses separately. Foremost, power is increased in case of the presence of genetic correlation between the different traits [44], but also multiple testing burden is alleviated compared to multiple univariate analyses. 

In this study we performed a multivariate GWAS using multivariate canonical correlation analysis (CCA) implemented in PLINK [45]. This method is based on extracting the linear combination of traits (i.e., metabolite levels and ratios) that explain the largest possible amount of covariation between the SNP and all traits. In comparison to other multivariate methods, it has been shown to perform particularly well in situations where both the phenotypes and their genetic control are correlated, as is expected in our data [46]. 

The traits included in our analysis are: PL in plasma, PL in CSF, PLP in plasma, PLP in CSF, PA in plasma, PLP:PL in plasma, PLP:PL in CSF, PA:PL in plasma, PA:PLP in plasma, PL in CSF:plasma and PLP in CSF:plasma, see Table 1. Prior to association, we regressed out covariates of sex, age and the first four MDS components to account for possible population stratification. We used the setting --mqfam-find 1 in order to retain individuals with missing data for certain phenotypes. Each association test yielded an F-statistic, a corresponding *p*-value, and a weight for each metabolite indicating the relative contribution of that metabolite to the overall association. To summarize these associations in terms of the index SNP with the highest association and other SNPs in high linkage disequilibrium with the index SNP, we used the following settings in PLINK: --clump-p1 1e-4 --clump-p2 1e-4 --clump-r2 0.1 --clump-kb 3000.

Human Genome 19 (UCSC Genome Browser) was used for SNP annotation. QQ- and Manhattan plots were generated with R and regional association plots were created using LocusZoom (http://statgen.sph.umich.edu/locuszoom/).

### 2.6. Univariate Analysis

In order to follow up our findings of interest, we also performed a univariate analysis for the phenotypes with the highest weights for our multivariate top hits (these are: PLP in CSF, PLP in plasma, PLP:PL in CSF, PLP:PL in plasma and PA:PLP in plasma). Univariate analysis was performed using PLINK (v1.08p) by applying an additive linear model on normalized metabolite levels including the same covariates of sex and age and four MDS components in the association. 

### 2.7. Gene-Based Association Using PrediXcan

Finally, we applied PrediXcan [47], a gene-based association method that prioritizes genes that are likely to be causal for a phenotype, to our data. The approach “imputes” gene expression from imputed genotype data, and by association with the phenotype it is able to identify genes involved in the etiology of the phenotype. 

We imputed the expression of 11,581 genes from our sample, using the prediction model DGN-WB_0.5.db [47], which is based on whole blood, and performed association analyses for all eleven included phenotypes. Without further correction for multiple testing, the threshold for genome-wide significant association was set to the Bonferroni-corrected *p*-value of 0.05/11,581 = 4.32 × 10^−6^.

## 3. Results 

Table 1 shows characteristics of the 493 genotyped subjects who remained after quality control, and their PL, PLP and PA concentrations and ratios in and between CSF and plasma (*n* = 399 for CSF, *n* = 480 for plasma and *n* = 386 for both). The B6 vitamer profile of CSF (PL > PLP) differed from that of plasma (PLP > PA > PL, *p* < 10 × 10^−16^ for each comparison, rank sum test). Concentrations of PLP in CSF were only 30% of those in plasma, whereas PL was almost three times higher in CSF than in plasma. 

**Figure 2:**
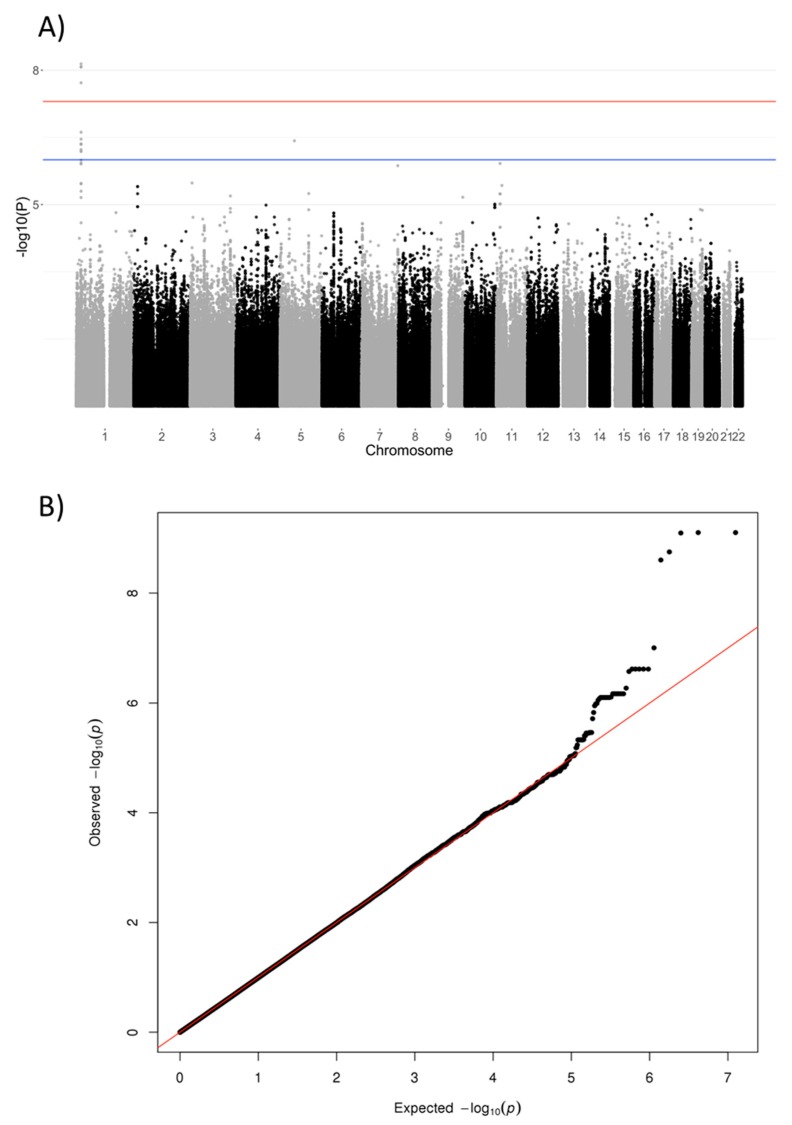
Manhattan plot of multivariate analysis of B6 metabolism in plasma and CSF (**A**). The red line indicates the genome-wide significance threshold of 5 × 10^−8^, the blue line 1 × 10^−6^. The QQ-plot of the association (**B**) indicating expected versus observed *p*-values. CSF: Cerebrospinal fluid; QQ-plot: Quantile-quantile-plot.

**Figure 3:**
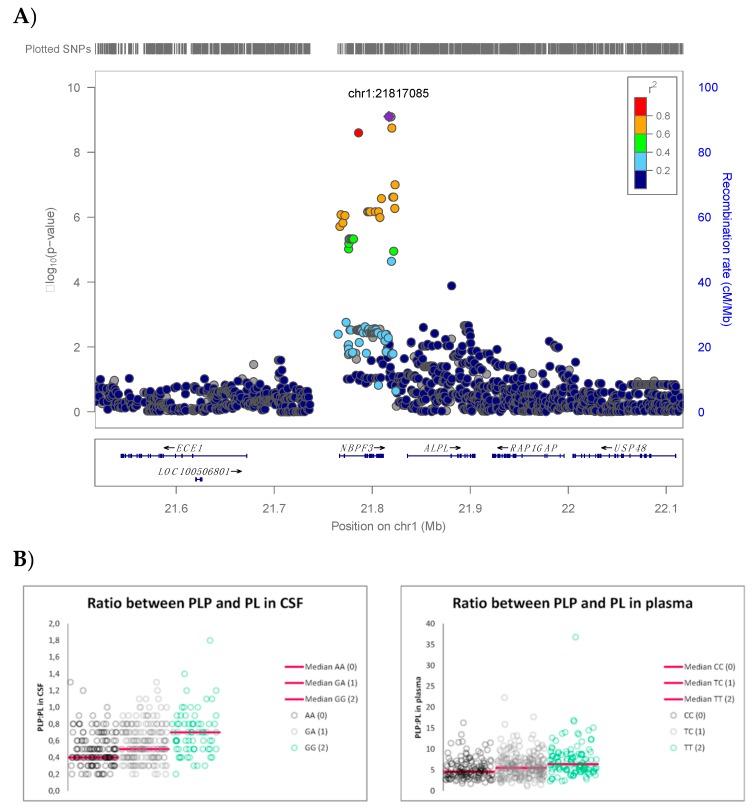
Regional association plot for the genome-wide significant locus for the ratio between PLP and PL on chromosome 1 (**A**) and scatterplots of the relation between genotype and measured PLP:PL in CSF (left) and plasma (right) (**B**).

Multivariate analysis resulted in one genome-wide significant locus on chromosome 1, containing the *NBPF3* (neuroblastoma breakpoint family, member 3) and *ALPL* (alkaline phosphatase) genes (Figure 2, Table 2, Figure 3A), with index SNP rs1106357 (*p* = 7.89 × 10^−10^, MAF = 0.46). This locus correlated mostly with the ratio between PLP and PL in plasma (weight 0.66) and the ratio between PLP and PL in CSF (weight 0.65) (Table 2; Supplementary Figure S2). Since the genomic inflation factor was 0.97, population stratification was not likely (Figure 2B). It should be noted that while the index SNP is imputed, the locus also contains a significant SNP that was genotyped (rs465474, *p* = 2.50 × 10^−9^). Univariate analysis of the phenotypes with the highest weights confirms this locus (Supplementary Table S2) and shows a univariate genome-wide significant association with the ratio between PLP and PL in CSF (*p* = 3.51 × 10^−9^) and with the ratio between PLP and PL in plasma (*p* = 4.16 × 10^−9^).

Subjects homozygous for the minor allele of rs1106357 showed a 1.6–times-higher ratio between PLP and PL in CSF (Figure 3B, Table 3) and a 1.4-times-higher ratio between PLP and PL in plasma than subjects homozygous for the major allele. In our sample, higher PLP:PL ratios in CSF and plasma are caused by higher concentrations of PLP rather than by lower PL: subjects homozygous for the minor allele showed a 1.5-times-higher concentration of PLP in CSF (20.3 vs. 13.9 nmol/L) and a 1.4-times-higher concentration of PLP in plasma (67.8 vs. 48.2 nmol/L) than subjects homozygous for the major allele of these SNPs. Concentrations of PL in CSF (29.8 vs. 30.4 nmol/L) and plasma (10.6 vs. 10.8 nmol/L) did not differ between genotypes for this SNP. Similarly, the plasma PA:PLP ratio in these subjects was 1.7 times lower (0.3 vs. 0.5) due to an increase of PLP, rather than due to altered concentrations of plasma PA (21.8 vs. 25.3 nmol/L). This suggests that the *ALPL* locus does not affect the degradation pathway of vitamin B6 to PA. 

Of additional interest is one locus on chromosome 15 (minimal *p* = 7.93 × 10^−7^, and MAF = 0.06 for rs28789220), which showed a suggestive association with the CSF:plasma ratio of PLP (weight 0.69) and to a lesser extent with the PLP:PL ratio in CSF (weight 0.39) (Supplementary Figure S3, Supplementary Table S2). The associated variants are located in an intron of an uncharacterized locus (*LOC102723493*). While all variants at this locus were imputed, imputation quality was very high (R^2^ 0.97–0.98). The univariate analysis of these variants with the CSF:plasma ratio of PLP also showed some association signal (*p* = 2.64 × 10^−6^), however, not reaching genome-wide significance (Supplementary Figure S4). 

None of the genes known to be involved in vitamin B6 metabolism (*PDXK*, *PNPO*, *PDXP* and *AOX1*) [24,25,26,27] showed any association (*p* > 1.0 × 10^−3^). 

The gene-based association using PrediXcan yielded no genes significantly associated with any of our included phenotypes. In addition, no genes at the significant locus on chromosome 1, the suggestive locus on chromosome 15, or any of the genes previously known to be involved in B6 metabolism showed suggestively significant associations (*p* < 1.0 × 10^−4^). Based on a lookup in the GTEx database, the top associated SNP rs1106357 on chromosome 1 is a significant expression quantitative trait locus (eQTL) for the gene for *NBPF3* in multiple tissues including liver, and to a lesser extent for *ALPL* in skin [48]. The variant on chromosome 15 is not a known eQTL for any gene. 

## 4. Discussion 

Here, we present the first GWAS of B6 vitamers and their ratios in and between CSF and plasma in a study of healthy adults. Despite the relatively small sample size, we were able to replicate a locus on chromosome 1 containing the *ALPL* gene (*p* = 7.89 × 10^−10^) previously associated with PLP and vitamin B6 in plasma [20,21,22,23] (Table 4). In our multivariate analyses, this locus was not only associated with plasma PLP and the plasma ratio between PLP and PL (weights 0.58 and 0.75, respectively), but at similar levels with PLP in CSF, and the CSF ratio between PLP and PL (weights of 0.59 and 0.62, respectively; Table 2). For these B6 vitamers and vitamer ratios, this locus reaches genome-wide significance by univariate analysis as well. Interestingly, the higher PLP:PL ratios in CSF and plasma are caused by higher concentrations of PLP and not by differences in PL. This finding not only replicates the association of plasma PLP concentrations with the *ALPL* locus, but shows that B6 metabolism in CSF is genetically regulated, and highlights the potential of genetic analyses of metabolites in CSF. 

The *ALPL* gene encodes a tissue-nonspecific and membrane-bound isoenzyme of alkaline phosphatase (ALPL; EC 3.1.3.1), which catalyzes hydrolysis reactions producing an alcohol and an inorganic phosphate from a phosphate monoester and water. Tissue expression of ALPL is ubiquitous, but liver, bone and kidney are the main locations for this enzyme. The physiological function of ALPL has not been fully elucidated, but its indispensable role in vitamin B6 uptake by cells (through hydrolysis of PLP into PL) is well known [19]. This role likely explains the strong association between the *ALPL* locus on chromosome 1 and the concentrations of PLP in CSF and plasma (Table 2). Since homozygosity for the minor allele is associated with higher PLP levels and PLP:PL ratios, rather than altered PL levels, we hypothesize that it is associated with decreased expression of *ALPL*. SNPs at the *ALPL* locus have indeed been shown to significantly influence plasma ALPL levels [49,50,51]. Unfortunately, our Predixcan analysis was not able to corroborate this hypothesis, possibly due to lack of power. In addition, the fact that the most significant SNP is an eQTL primarily for neuroblastoma breakpoint family member 3 (*NBPF3*), the other gene at the locus, rather than *ALPL*, leaves room for further investigation. Prior to our GWAS analyses, we normalized B6 vitamer levels by applying rank inverse transformation. Normalization of the phenotype is necessary to perform a linear regression, but it may affect the exact reported effect sizes in our study, as well as others. However, in the present study, the reported effect sizes are strong and robust. 

In our study we have not only measured PLP and PL as in previous studies [20,21,22,23], but the degradation product PA as well. The absence of any link with PA levels suggests indeed that ALPL plays a role in B6 uptake rather than in vitamin B6 degradation. 

Limited by current sample sizes, our study is underpowered to fully disentangle the genetic contribution of vitamin B6 metabolism in CSF versus that in plasma. For example, the regulation of B6 levels through the *ALPL* locus may be independent in plasma and CSF, or the observed signals may both be due to genetic regulation in the liver. However, the suggestive signal on chromosome 15, while not reaching genome-wide significance, is of particular interest. This locus is associated specifically with the ratio of PLP between CSF and plasma, and may be involved with vitamin B6 transport across the blood–brain or blood–CSF barriers. Future studies with larger sample sizes are needed to corroborate this hypothesis, and are likely to elucidate biological aspects underlying vitamin B6 generation, transport and degradation.

## Figures and Tables

**Table 1:** Characteristics of the 493 genotyped subjects and their B6 vitamer (pyridoxal (PL), pyridoxal phosphate (PLP)) and pyridoxic acid (PA) concentrations (nmol/L) and ratios in and between cerebrospinal fluid (CSF) and plasma.

		Number	Median	Range
**Sex**		353 male 140 female	n.a.	n.a.
**Age (years)**		493	42	18–63
**PL**	**Plasma**	480	10.5	3.0–56.2
	**CSF**	399	30.0	13.5–66.4
**PLP**	**Plasma**	480	55.7	10.2–335
	**CSF**	399	16.0	5.3–45.8
**PA**	**Plasma**	480	23.6	2.7–243
**PLP:PL**	**Plasma**	480	5.4	1.1–36.8
	**CSF**	399	0.5	0.2–1.8
**PA:PL**	**Plasma**	480	2.4	0.3–15.1
**PA:PLP**	**Plasma**	480	0.44	0.03–2.5
**PL in CSF:plasma**		386	2.9	0.9–10.6
**PLP in CSF:plasma**		386	0.3	0.1–0.9

**Table 2:** Genome-wide significant locus for B6 vitamer (PL, PLP) and PA concentrations and ratios in and between CSF and plasma (*n* = 493). Only weights ≤−0.30 or ≥0.30 are shown.

*Index* SNP	Chr	Allele	Position (bp)	MAF	Genes	F	Significance (*p*-Value)	OMIM	Weights				
									PLP in CSF	PLP in Plasma	PLP:PL in CSF	PLP:PL in Plasma	PA:PLP in Plasma
rs1106357	1	T/C	2,181,7085	0.46	*NBPF3*, *ALPL*	6.34	7.89 × 10^−10^	#146300 #241500 #241510	0.58	0.48	0.65	0.66	−0.58

SNP = single nucleotide polymorphism, Chr = chromosome, bp = base pair (HG19), MAF = minor allele frequency, F = F statistic.

**Table 3:** Relation between genotype and phenotype for the genome-wide significant locus. Only phenotypes with the highest weight for each genotype are shown.

SNP	Chr	Genotype (*n*)	PLP:PL in Plasma (Median (Range))	Significance (*p*-Value) *	PLP:PL in CSF (Median (Range))	Significance (*p*-Value) *
rs1106357	1	0 (143)	4.6 (1.7–16.3)	0 vs. 1 3.23 × 10^−3^ 0 vs. 2 <5.0 × 10^−7^ 1 vs. 2 5.95 × 10^−4^	0.4 (0.2–1.3)	0 vs. 1 3.26 × 10^−3^ 0 vs. 2 <5.0 × 10^−7^ 1 vs. 2 4.45 × 10^−4^
		1 (233)	5.5 (1.1–22.3)		0.5 (0.2–1.3)	
		2 (104)	6.4 (2.3–36.8)		0.7 (0.2–1.8)	

Genotype: 0 = 0 minor alleles, 1 = 1 minor allele, 2 = 2 minor alleles; *n* = number; * = Mann-Whitney U test.

**Table 4:** Published genome-wide significant associations for plasma vitamin B6 (PLP).

Index SNP	Chr	Allele	Position (bp)	MAF	Beta *	Significance (*p*-Value)	Body Fluid & B6 Vitamer	Methods	Reference
rs1256335	1	A/G	21,890,386	0.21	−0.14	1.40 × 10^−15^	Plasma PLP	Meta-analysis of three GWAS (*n* = 4763)	[22]
rs4654748		C/T	21,786,068	0.50	−1.45	8.30 × 10^−18^	Plasma vitamin B6	Four GWAS cohort meta-analysis (*n* = 1864)	[23]
rs1697421		G/A	21,823,292	0.47	0.173	7.06 × 10^−10^	Plasma PLP	GWAS (*n* = 2100)	[20]
rs1772719		A/C	21,904,374	0.23	−0.06	2.48 × 10^−16^	Plasma PLP	GWAS (*n* = 2158)	[21]

* For Hazra et al. [22], the beta is generated using a fixed-effects model after log-transformation of plasma PLP concentrations; for Tanaka et al. [23], the beta represents the change in amount of vitamin B6 (ng/mL) per copy of the C allele of the SNP; for Keene et al. [20], the beta represents the linear regression coefficient after inverse normal transformation of plasma PLP concentrations in an additive model including age, sex and the top 10 principal components; for Carter et al. [21], the beta is based on a linear regression model adjusted for age, sex and vitamin B6 intake, and the outcome variable is a log10-transformation of the plasma PLP concentration. All reported associations are at the *ALPL*/*NBPF3* locus on chromosome 1.

## Supplementary Materials

The following are available online at http://www.mdpi.com/2073-4425/10/1/8/s1. Table S1: Univariate *p*-values for SNPs with genome-wide significant multivariate associations for B6 vitamer (PL, PLP) and PA concentrations and ratios in and between CSF and plasma; Table S2: Loci with suggestively significant associations for B6 vitamer (PL, PLP) and PA concentrations and ratios in and between CSF and plasma; Figure S1: MDS plot based on a set of 87,956 independent high-quality SNPs using our sample (A) and clustered using HapMap3 (B); Figure S2: Correlation between PLP and PL in CSF and plasma; Figure S3: Regional association plot for the suggestive (but nonsignificant) locus on chromosome 15. Figure S4: Manhattan plot for the univariate analysis of the CSF:plasma ratio of PLP.
