# Supplementary material for: The Alkaline Phosphatase (ALPL) Locus Is Associated with B6 Vitamer Levels in CSF and Plasma
Source: Genes (Basel). 2018 Dec 22;10(1):8. doi: 10.3390/genes10010008 (PMC6357176; doi:10.3390/genes10010008)
Supplement: Supplementary file 1 [file genes-10-00008-s001.zip › genes-410552-supplementary-figures-final.docx]

Supplemental Figures: The alkaline phosphatase (*ALPL*) locus is associated with B6 vitamer levels in CSF and plasma Olde Loohuis and Albersen et al.

A.

B.

**Supplementary Figure 1.** MDS plot based on a set of 87,956 independent high quality SNPs using our sample (A) and clustered using HapMap3 (B). Based on B we excluded two ethnic outliers indicated by black arrows.

**Supplementary Figure 2**: Correlation between PLP and PL in CSF (left) and plasma (right).

**Supplementary Figure 3:** Regional association plot for the suggestive (but non-significant) locus on chromosome 15.

**Supplementary Figure 4:** Manhattan plot of univariate analysis of PLP in plasma:CSF. The red line indicates the genome-wide significance threshold of 5 x 10^-08^, the blue line 1x 10^-06^.
