# Supplementary material for: The Alkaline Phosphatase (ALPL) Locus Is Associated with B6 Vitamer Levels in CSF and Plasma
Source: Genes (Basel). 2018 Dec 22;10(1):8. doi: 10.3390/genes10010008 (PMC6357176; doi:10.3390/genes10010008)
Supplement: Supplementary file 1 [file genes-10-00008-s001.zip › genes-410552-supplementary-tables-final.docx]

**Supplementary Table 1** Univariate p-values for SNPs with genome-wide significant multivariate associations for B6 vitamer (PL, PLP) and PA concentrations and ratios in and between CSF and plasma (*n*=493).

| SNP | Chr | Allele | Position (bp) | MAF | Gene | OMIM | Significance (p-value) [Beta] | | | | |
| --- | --- | --- | --- | --- | --- | --- | --- | --- | --- | --- | --- |
|  |  |  |  |  |  |  | PLP in CSF | PLP in plasma | PLP:PL in CSF | PLP:PL in plasma | PA:PLP in plasma |
| rs1106357 | 1 | T/C | 21817085 | 0.46 | *NBPF3* (downstream) *ALPL* (upstream) | #146300 #241500 #241510 | 3.11E-06 [0.322] | >1.0E-04 [na] | 1.48E-07 [0.349] | 1.40E-07 [0.331] | 3.13E-06 [-0.288] |
| rs80212518 |  | GGT/G | 21817126 | 0.46 |  |  | 3.11E-06 [0.322] | >1.0E-04 [na] | 1.48E-07 [0.349] | 1.40E-07 [0.331] | 3.13E-06 [-0.288] |
| rs201680459 |  | G/GT | 21819159 | 0.46 |  |  | 3.03E-06 [0.324] | >1.0E-04 [na] | 9.89E-08 [0.355] | 1.28E-07 [0.333] | 5.00E-06 [-0.283] |
| rs12132412 |  | G/A | 21820042 | 0.41 |  |  | 1.56E-07 [0.366] | >1.0E-04 [na] | 3.51E-09 [0.396] | 1.35E-06 [0.310] | 1.40E-05 [-0.274] |
| rs4654748 % |  | T/C | 21786068 | 0.48 | *NBPF3* (intronic) |  | 3.35E-06 [0.317] | 5.77E-06 [0.280] | 1.01E-06 [0.321] | 4.16E-09 [0.368] | 2.65E-05 [-0.260] |

SNP = single nucleotide polymorphism, Chr = chromosome, bp = base pair (HG19), MAF = minor allele frequency, % = published SNP (see Table 4), na = not applicable

**Supplementary Table 2** Loci with suggestively significant associations for B6 vitamer (PL, PLP) and PA concentrations and ratios in and between CSF and plasma (*n*=493). Only weights ≤ -0.30 or ≥ 0.30 are shown.

| Index **SNP** | **Chr** | **Allele** | **Position (bp)** | **MAF** | **Gene** | **F** | **Significance (p-value)** | **Weights** | | | | | | | |
| --- | --- | --- | --- | --- | --- | --- | --- | --- | --- | --- | --- | --- | --- | --- | --- |
|  |  |  |  |  |  |  |  | **PLP in CSF** | **PLP in plasma** | | **PLP:PL in CSF** | | | **PLP:PL in plasma** | **PA:PLP in plasma** |
| **rs1697421 %** | 1 | C/T | 21823292 | 0.49 | NBPF3 , ALPL | 5.19 | 9.97E-08 | 0.66 | 0.54 | | **0.68** | | | 0.66 | -0.50 |
| **SNP** | **Chr** | **Allele** | **Position (bp)** | **MAF** | **Gene** | **F** | **Significance (p-value)** | **Weights** | | | | | | | |
|  |  |  |  |  |  |  |  | **PL in CSF** | **PLP in plasma** | **PLP:PL in plasma** | | | **PA:PLP in plasma** | **PL in CSF: plasma** | **PLP in CSF: plasma** |
| **rs10056527** | 5 | T/C /T/C | 124076083 | 0.22 | ZNF608 (intronic) | 4.69 | 7.79E-07 | 0.48 | 0.53 | 0.63 | | | 0.50 | 0.32 | **0.78** |
| **SNP** | **Chr** | **Allele** | **Position (bp)** | **MAF** | **Gene** | **F** | **Significance (p-value)** | **Weights** | | | | | | | |
|  |  |  |  |  |  |  |  | **PLP:PL in CSF** | | | | **PLP in CSF:plasma** | | | |
| **rs28789220** | 15 | C/G | 67280928 | 0.06 | LOC102723493 (intronic) | 4.69 | 7.93E-07 | 0.39 | | | | **0.69** | | | |

SNP = single nucleotide polymorphism, Chr = chromosome, bp = base pair (HG19), MAF = minor allele frequency, F = F statistic, % = published SNP (see Table 4)
